# Spontaneous aspiration of a long tree twig as foreign body

**DOI:** 10.1002/rcr2.401

**Published:** 2019-02-01

**Authors:** Kosuke Hashimoto, Kyoichi Kaira, Kunihiko Kobayashi, Yoshitake Murayama, Hiroshi Kagamu

**Affiliations:** ^1^ Department of Respiratory Medicine Comprehensive Cancer Center, International Medical Center, Saitama University Hospital Saitama Japan

**Keywords:** Foreign body, long tree twig spontaneous aspiration, tracheal stoma

## Abstract

Spontaneous aspiration of a long tree twig as foreign body is an extremely rare condition. The presence of a permanent tracheal stoma in a laryngectomized patient should be considered as a predisposing factor for foreign body aspiration.

## Clinical Image

**Figure 1:**
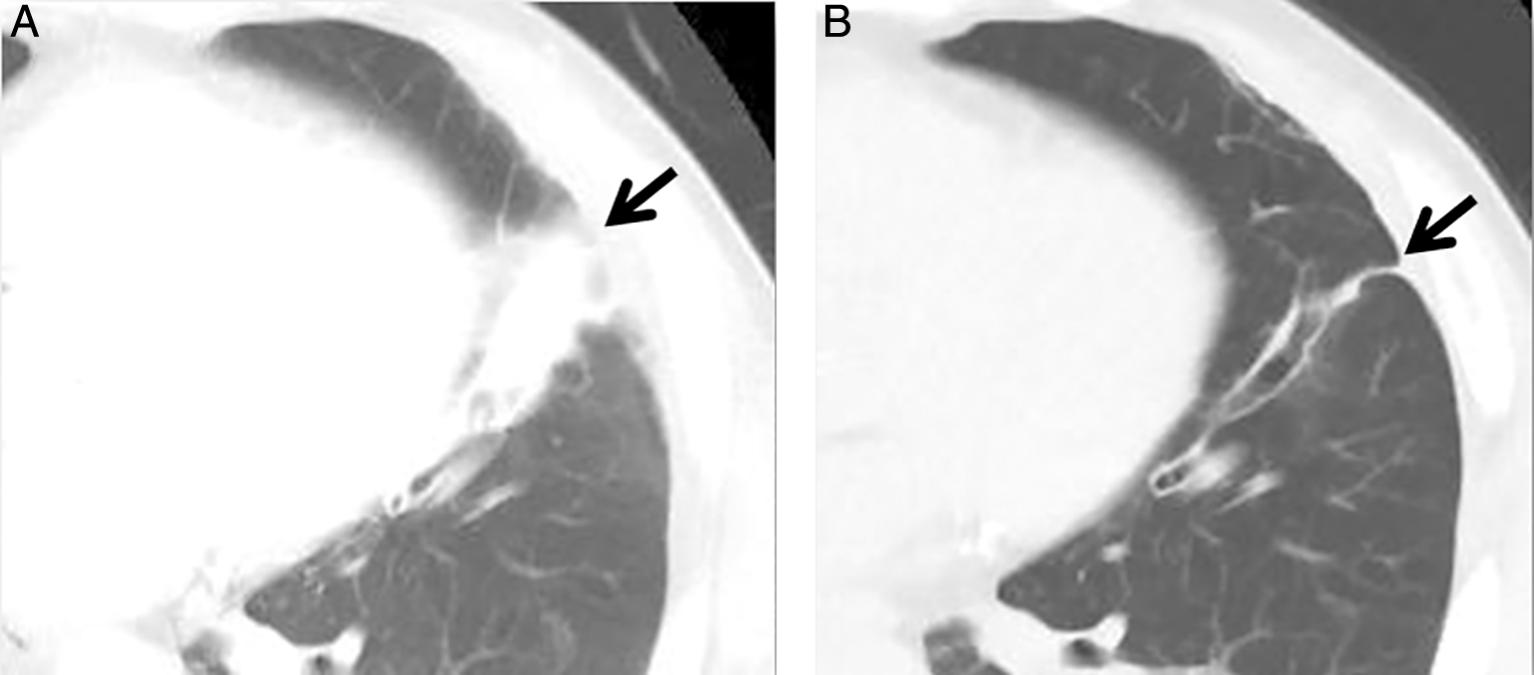
Chest computed tomography (CT) after aspiration of a tree twig: CT scan after aspiration of foreign body reveals consolidation in the lingular segment (A) (black arrow), but this consolidation improves after removal of foreign body (B) (black arrow).

**Figure 2:**
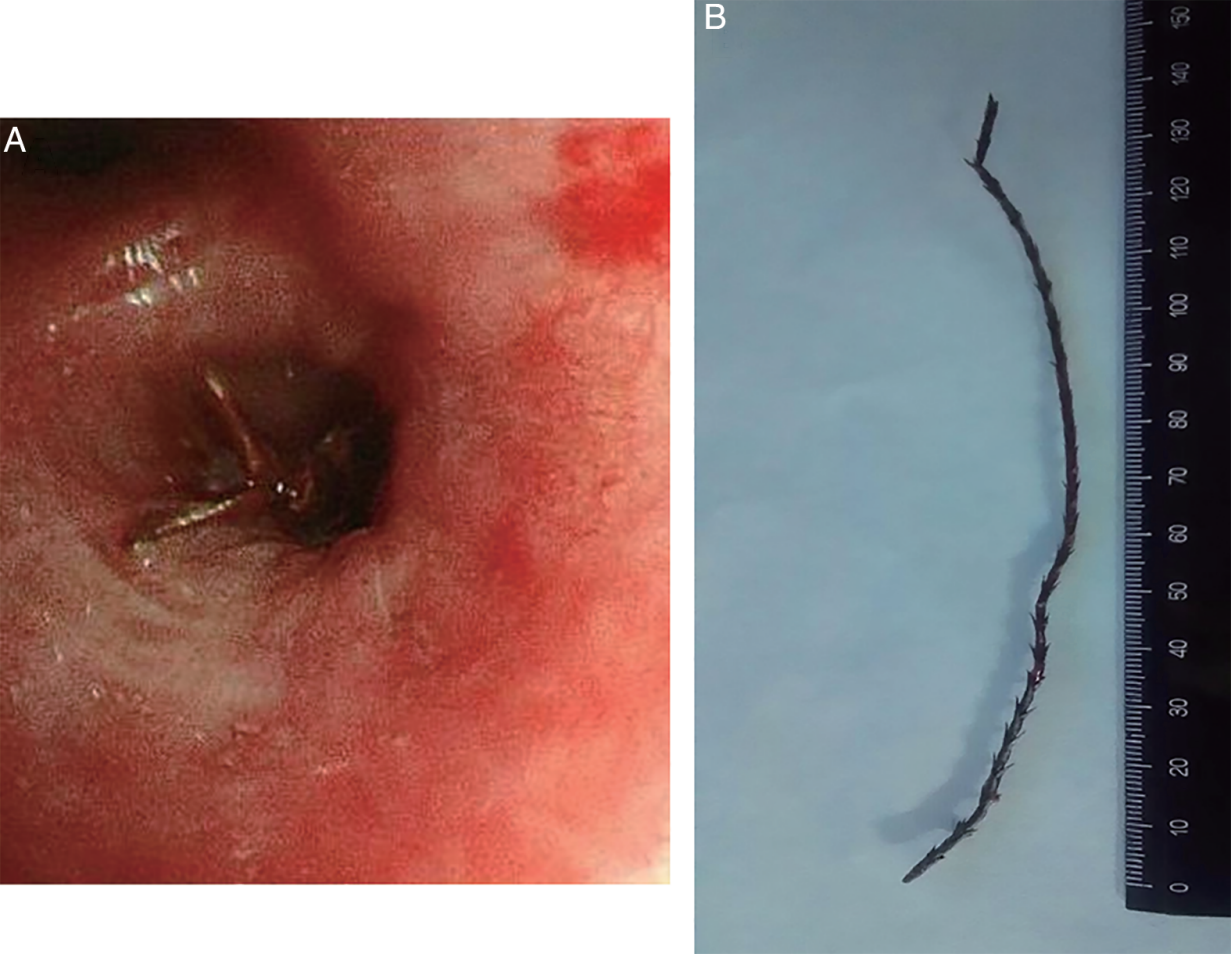
A bronchoscopic finding shows a dark brown rigid foreign body in the inlet of lingular segment (A), and a tree twig with a diameter of 140 mm was removed by bronchoscopy (B).

A 73‐year‐old man underwent total laryngectomy for left supraglottic cancer, after which a permanent tracheal stoma was made. While pruning cypress offshoots without a tracheal pit filter 4 months ago, he developed a sudden cough and haemosputum, with no self‐awareness of aspiration. These symptoms disappeared spontaneously; however, he was referred to our department because of an abnormal chest shadow on X‐ray images. The chest radiograph showed an infiltrative shadow in the left lower field and computed tomography (CT) of the chest also showed consolidation in the lingular segment (Fig. 1A). Although physical examination, including neurological examination and laboratory tests, was unremarkable, a diagnostic bronchoscopy was performed to exclude the possibility of recurrent supraglottic cancer. The bronchoscopic finding revealed a dark brown rigid foreign body in the inlet of the lingular segment (Fig. 2A). We attempted to pull out the foreign body, and the matter itself was completely removed with the bronchoscope. A 140‐mm diameter tree twig was identified (Fig. 2B). The foreign body was consistent with the cypress that he was pruning. Chest CT also showed that the pulmonary abnormality improved after removal of the foreign body (Fig. 1B).

In our case, the patient took off the tracheal pit filter, thus allowing a tree twig to accidentally reach the segmental bronchus through the tracheal stoma without self‐awareness. This is not a clinical scenario that is usually considered by clinicians. At the initial examination, nobody was aware of foreign body aspiration, and we considered that the pulmonary infiltrative opacity was possibly secondary to other reasons such as bacterial infection or recurrence of cancer. A diagnostic bronchoscopy was useful to discover the presence of a tree twig following foreign body aspiration. Foreign body aspiration into the tracheobronchial tree is extremely rare in adults, and the presence of a permanent tracheal stoma in a laryngectomized patient is important as a predisposing factor for foreign body aspiration. Several researchers have reported that the presence of such stoma may be related to foreign body aspiration in patients with a permanent laryngectomized tracheal stoma 1, 2. To our knowledge, however, there are no reports about the aspiration of a tree twig that was initially noticed as an abnormal pulmonary shadow despite having no neurological disorder. As a possible mechanism by which a tree twig may be aspirated into the peripheral bronchus, we suggest that the twig easily entered the bronchus and remained there, since a tree twig has regular barbs akin to the teeth of a saw.

Although accidental aspirations such as that in our present case do not occur in patients without tracheal stoma, physicians should be alert to the possibility of a foreign body when any pulmonary infiltrative opacity is seen in a laryngectomized patient with tracheal stoma.

### Disclosure Statement

Appropriate written informed consent was obtained for publication of this case report and accompanying images.
